# Publisher Correction: Control of laser plasma accelerated electrons for light sources

**DOI:** 10.1038/s41467-018-04299-1

**Published:** 2018-05-02

**Authors:** T. André, I. A. Andriyash, A. Loulergue, M. Labat, E. Roussel, A. Ghaith, M. Khojoyan, C. Thaury, M. Valléau, F. Briquez, F. Marteau, K. Tavakoli, P. N’Gotta, Y. Dietrich, G. Lambert, V. Malka, C. Benabderrahmane, J. Vétéran, L. Chapuis, T. El Ajjouri, M. Sebdaoui, N. Hubert, O. Marcouillé, P. Berteaud, N. Leclercq, M. El Ajjouri, P. Rommeluère, F. Bouvet, J. -P. Duval, C. Kitegi, F. Blache, B. Mahieu, S. Corde, J. Gautier, K. Ta Phuoc, J. P. Goddet, A. Lestrade, C. Herbeaux, C. Évain, C. Szwaj, S. Bielawski, A. Tafzi, P. Rousseau, S. Smartsev, F. Polack, D. Dennetière, C. Bourassin-Bouchet, C. De Oliveira, M. -E. Couprie

**Affiliations:** 1 Synchrotron-SOLEIL, L’Orme des Merisiers, Saint-Aubin, 91192 France; 2 Université Paris-Saclay, Paris, 91190 France; 3 PhLAM, UMR CNRS 8523, Université Lille 1, Sciences et Technologies, 59655 Villeneuve d’Ascq, France; 4 LOA, École polytechnique, ENSTA ParisTech, CNRS, Université Paris-Saclay, 828 Bd des Maréchaux, 91762 Palaiseau Cedex, France; 5 Department of Physics of Complex Systems, Weizmann Institute of Science, Rehovot, 761001 Israel

Correction to: *Nature Communications* 10.1038/s41467-018-03776-x; published online: 06 April 2018

The original version of this Article contained an error in the last sentence of the first paragraph of the Introduction and incorrectly read ‘A proper electron beam control is one of the main challenges towards the Graal of developing a compact alternative of X-ray free-electron lasers by coupling LWFA gigaelectron-volts per centimetre acceleration gradient with undulators in the amplification regime in equation 11, **n**x(**n**-**β**) x **β**: n the two times and beta the two times should be bold since they are vectorsin Eq. 12, **β** should be bold as well.’

The correct version is ‘A proper electron beam control is one of the main challenges towards the Graal of developing a compact alternative of X-ray free-electron lasers by coupling LWFA gigaelectron-volts per centimetre acceleration gradient with undulators in the amplification regime.’

This has been corrected in both the PDF and HTML versions of the Article.

